# From Youthful Vigor to Aging Decline: Unravelling the Intrinsic and Extrinsic Determinants of Hippocampal Neural Stem Cell Aging

**DOI:** 10.3390/cells12162086

**Published:** 2023-08-17

**Authors:** Patricia Jiménez Peinado, Anja Urbach

**Affiliations:** 1 Department of Neurology, Jena University Hospital, 07747 Jena, Germany; 2 Jena Center for Healthy Aging, Jena University Hospital, 07747 Jena, Germany; 3 Aging Research Center Jena, Leibniz Institute on Aging, 07745 Jena, Germany

**Keywords:** aging, adult hippocampal neurogenesis, adult neural stem cells, intrinsic mechanisms, neurogenic niche, systemic environment, rejuvenation

## Abstract

Since Joseph Altman published his pioneering work demonstrating neurogenesis in the hippocampus of adult rats, the number of publications in this field increased exponentially. Today, we know that the adult hippocampus harbors a pool of adult neural stem cells (NSCs) that are the source of life-long neurogenesis and plasticity. The functions of these NSCs are regulated by extrinsic cues arising from neighboring cells and the systemic environment. However, this tight regulation is subject to imbalance with age, resulting in a decline in adult NSCs and neurogenesis, which contributes to the progressive deterioration of hippocampus-related cognitive functions. Despite extensive investigation, the mechanisms underlying this age-related decline in neurogenesis are only incompletely understood, but appear to include an increase in NSC quiescence, changes in differentiation patterns, and NSC exhaustion. In this review, we summarize recent work that has improved our knowledge of hippocampal NSC aging, focusing on NSC-intrinsic mechanisms as well as cellular and molecular changes in the niche and systemic environment that might be involved in the age-related decline in NSC functions. Additionally, we identify future directions that may advance our understanding of NSC aging and the concomitant loss of hippocampal neurogenesis and plasticity.

## 1. Introduction

Contrary to the long-held belief that neurogenesis tapers off with the end of early postnatal development, the mammalian brain retains the capacity to generate new neurons throughout life. This process, termed adult neurogenesis, is mainly restricted to two specific brain regions, the subventricular zone (SVZ) of the lateral ventricles and the subgranular zone (SGZ) of the dentate gyrus (DG) in the hippocampus [1,2].

**Figure 1:**
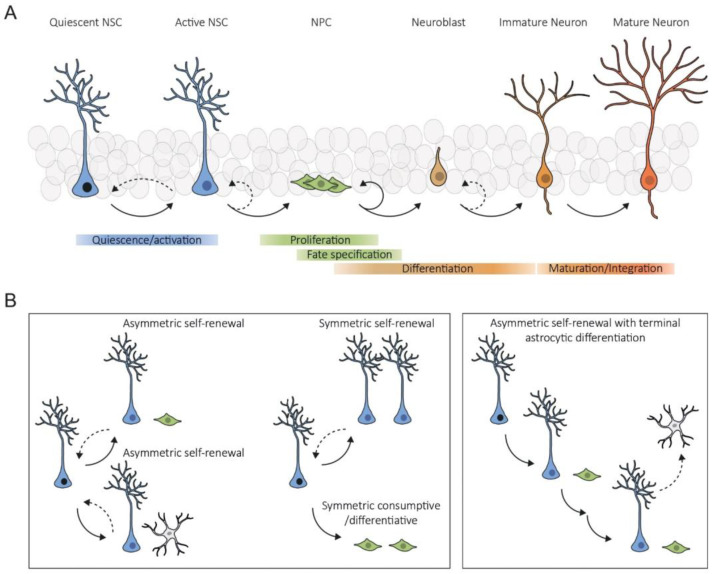
Neurogenesis in the adult hippocampus. (**A**) Generation of mature granule cell neurons from NSCs is a multi-step process involving various stages and processes. (**B**) Distinct division modes of NSCs in the adult SGZ. When a quiescent NSC is activated, it preferentially divides asymmetrically to self-renew and produce a NPC or, rarely, an astrocyte. It can also divide symmetrically to expand the pool or to produce two NPCs. Moreover, while some NSCs possess long-term self-renewal potential and return to quiescence after division (dashed arrows in **left panel**), others have limited self-renewal potential and differentiate into astrocytes after several rounds of division, thus becoming depleted from the pool (**right panel**).

The basis of adult hippocampal neurogenesis lies in a population of self-renewing and multipotent NSCs (Figure 1) residing in a specialized microenvironment known as neurogenic niche [3,4]. During most of their lifespan, NSCs are quiescent, a reversible state in which they do not divide but maintain the capacity to re-enter the cell cycle. When activated, these cells can produce progeny by both asymmetric and symmetric division, which may either maintain (asymmetric self-renewal), expand (symmetric self-renewal), or deplete (symmetric consumptive/differentiative) the NSC pool (Figure 1). Most divisions in the SGZ are asymmetric [4,5,6] and give rise to a daughter NSC and a neural progenitor cell (NPC), which generates neurons that functionally integrate into the hippocampal circuitry (Figure 1A) and contribute to hippocampus-related cognitive functions such as episodic memory and mood regulation [7]. Recent data suggest that the nature and rate of NSC divisions can be markedly heterogeneous depending on their history, the stimulus, and age (Figure 1B) [5,8,9,10,11,12,13]. Moreover, while some NSCs may possess long-term self-renewal potential and return to quiescence after division, others have limited self-renewal potential and, once activated, become depleted from the NSC pool via exhaustion and terminal differentiation [4,5,6,10]. This shows that maintaining a balance between NSC quiescence and activation is critical to prevent their depletion and to preserve a pool of functional NSCs for life-long neurogenesis. This balance is achieved by an intricate interplay between intrinsic factors and extrinsic signals originating from local niche cells and from the systemic environment that collectively control the behavior of NSCs [14,15].

Adult hippocampal neurogenesis declines markedly with advancing age, which contributes to cognitive deterioration and neurodegenerative diseases [16,17,18,19,20]. Although the precise mechanisms underlying this age-related decline are still not well understood, evidence points to a numerical and functional loss of NSCs. This likely involves increased NSC quiescence, changes in division mode, a progressive loss of stem cell properties, NSC exhaustion, terminal differentiation into astrocytes, and an increase in NSC senescence, resulting in fewer NPCs and neurons being produced [5,9,11,21,22,23]. In addition, aging affects other stages of adult hippocampal neurogenesis, which includes, for example, lower proliferation and neuronal differentiation of progenitor cells or reduced survival of newly born neurons [24]. Moreover, both the cellular composition of the niche as well as the signals derived from the local and systemic environments undergo extensive changes with age [25,26,27]. However, it is still unclear how exactly changes in the niche and those in the NSCs themselves contribute to NSC aging and the decline in adult hippocampal neurogenesis.

In this review, we will summarize the current state of knowledge of the intrinsic mechanisms associated with hippocampal NSC aging, such as impaired metabolism and mitochondrial function, dysregulation of protein homeostasis, DNA damage and epigenetic alterations, and cellular senescence. We will also address the molecular and cellular changes in the neurogenic niche and systemic environment that might contribute to the age-related decline in NSC functions. If little or no information exists for the SGZ, we discuss findings in SVZ-NSCs or other somatic stem cells to derive concepts for stem cell aging in the SGZ.

## 2. Intrinsic Mechanisms

**Figure 2:**
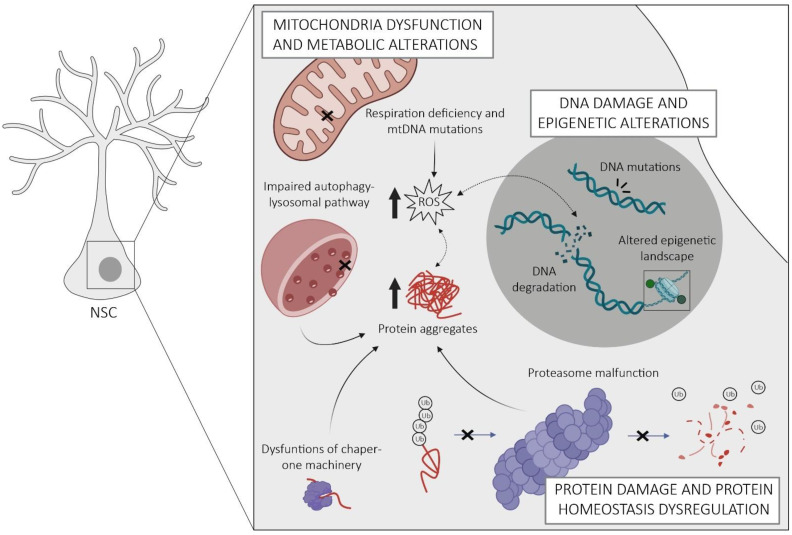
Schematic representation of the key intrinsic mechanisms of NSC aging in the SGZ. Aged NSCs are characterized by mitochondrial dysfunction leading to the accumulation of reactive oxygen species (ROS) and oxidative stress. This causes damage to cellular components such as proteins and DNA, which entails the formation of protein aggregates and the accumulation of DNA mutations. Despite this, aged NSCs exhibit a dysregulated protein homeostasis arising from malfunctions of the proteasome, chaperones, and autophagy–lysosomal pathways. Changes in their epigenetic landscape lead to alterations in gene expression patterns. Notes: the non-dashed and dashed arrows indicate unidirectional and bidirectional relationships, respectively. Crosses indicate a malfunction.

The regulation of NSCs is inherently complex and crucial for maintaining their regenerative potential throughout life. Within these cells, there exist intricate and interconnected mechanisms that homeostatically regulate their molecular, structural, and functional properties, which become dysregulated with age, adversely affecting the functions of NSCs. In this section, we provide an overview of the currently known intrinsic mechanisms underlying NSC aging, encompassing alterations in mitochondrial function and metabolism, disturbances in protein homeostasis, DNA damage, and alterations in epigenetic patterns, as well as the onset of cellular senescence (Figure 2).

### 2.1. Protein Homeostasis Dysregulation

The accumulation of misfolded or damaged proteins is a critical determinant of stem cell proliferation and aging [28]. Similar to other stem cells, adult NSCs use multiple interconnected pathways to regulate the balance of intracellular proteins (proteostasis), including molecular chaperones, the autophagy–lysosomal pathway, and the proteasome. Chaperones play a central role at all levels of proteostasis by facilitating proper folding of nascent or damaged proteins, assisting in protein translocation and stabilization, preventing protein aggregation, and supporting protein degradation via the ubiquitin–proteasome system and autophagy [28,29]. The proteasome is responsible for the degradation of irreversibly misfolded or unneeded proteins [30]. Therefore, it serves not only to prevent protein aggregation but also plays a critical role in timely removal of many regulatory proteins, such as those involved in the cell cycle, gene expression, and stress responses [28]. The major task of the autophagy–lysosomal pathway in proteostasis is the clearance and degradation of deleterious protein aggregates [31].

Active and quiescent NSCs rely on different pathways to maintain homeostasis in their proteome. Whereas active NSCs display active proteasomes, quiescent NSCs accumulate aggregates in larger lysosomes [32,33]. Notably, these differences determine the fate of NSCs. For example, abrogation of lysosomal activity by chemical inhibition or by deletion of the lysosomal master regulator transcription factor EB (TFEB) resulted in activation of quiescent NSCs and delayed return to quiescence in active NSCs, indicating a requirement of lysosomal activity for maintaining NSC quiescence [33]. Furthermore, active and quiescent NSCs utilize different types of chaperones. Active NSCs express higher amounts of endoplasmic reticulum (ER) unfolded protein response genes responsible for maintaining proteostasis in the ER when newly synthesized protein load exceeds the folding capacity or upon stress-induced accumulation of misfolded proteins [32]. In contrast, quiescent NSCs exhibit increased expression of T-complex protein 1 (TCP-1) ring complex and its upstream prefoldin complex, which maintain misfolded protein solubility and provide stress resilience in adult NSCs [32,34].

Proteostasis capacity declines in aged NSCs, leading to the accumulation of protein aggregates, impaired self-renewal, and lower resilience to stress [32,34,35]. This mainly concerns clearance mechanisms enriched in quiescent NSCs, including a decrease in TCP-1 ring complex and impairments in the autophagy–lysosomal pathway [32,34]. The mechanisms responsible for these alterations are not well understood but may involve members of the class O of forkhead box transcription factors (FoxOs), which are transcriptional activators of genes involved in stress-inducible chaperone response, lysosome-autophagy, and the proteasome [29,35,36]. FoxOs participate in nutrient-sensing pathways and activate when the insulin/insulin-like-growth-factor-I (IGF-1) signaling is low, resulting in enhanced autophagy-mediated protein clearance [29]. Accordingly, deletion of FoxOs in adult NSCs, which impairs autophagy and leads to accumulation of damaged proteins, is associated with hyperproliferation and subsequent depletion of hippocampal NSCs [35,37]. This highlights the importance of FoxO-mediated autophagy in restricting the activation of young NSCs to prevent their premature exhaustion [37]. Conversely, in aged NSCs, autophagy becomes necessary for their activation from quiescence. Here, it has been shown that increasing autophagy, for example by overexpressing TFEB or fasting, alleviates the proliferation deficit of aged NSCs [32]. Limited knowledge exists on age-related changes in proteasome activity in hippocampal NSCs. A recent study suggests that NSCs in the SVZ preserve proteasome activity throughout their lifespan [32], in contrast with somatic cells that experience dysfunctional proteasomal function with age [28]. Overall, available evidence suggests that defective lysosomal degradation plays a major role in NSC aging. Yet, further studies are needed to elucidate the specific mechanisms linking impaired autophagy to NSC dysfunction and their potential implications for preserving their regenerative potential during aging. These include, for example, dysregulation of mitochondria, ROS production, and DNA integrity, which present common points of convergence for autophagy alterations in other stem cell contexts. Considering the dynamic nature of proteostasis and its susceptibility to external factors, interventions such as dietary restriction hold promise in mitigating age-related decline in hippocampal NSCs.

Loss of proteostasis is exacerbated by defective segregation of damaged proteins during asymmetric division of aged NSCs. Similar to other somatic stem cells, NSCs possess mechanisms to prevent the retention of dysfunctional proteins and other harmful cargo during division. This includes a diffusion barrier within the ER membranes that facilitates the segregation of damaged proteins to their differentiating progeny [38]. However, aging diminishes the efficiency of this barrier so that damaged proteins are passed more evenly, leading to an accumulation of aging factors in aged NSCs with potentially detrimental consequences for their proliferative capacity [38]. The functionality of this barrier is believed to involve lamins A and B1, components of the nuclear lamina that integrate into the endoplasmic membrane during cell division [38,39]. Evidence suggests that decreased expression of lamins and the accumulation of incorrectly processed lamin precursor proteins contribute to barrier weakening with aging [38,39,40]. Furthermore, given their role in stem cell proliferation and fate determination, altered levels of lamins in aging are likely to cause increased quiescence and astrocytic differentiation of aged NSCs [5,38,39,41,42]. Yet, lamins have diverse nuclear and cellular functions, ranging from chromatin organization and gene regulation to cytoskeletal organization [43]. Therefore, further investigation is warranted to establish a causal relationship between their role in barrier strength and the protection of NSCs against aging.

In summary, the dysregulation of proteostasis during aging disrupts the mechanisms that maintain NSC functions and leads to the accumulation of dysfunctional proteins. This can induce cellular stress and inflammatory responses, and impairments in NSC self-renewal and maintenance, which contribute to reduced neurogenesis and compromised plasticity in the aged hippocampus. Understanding and targeting altered proteostasis mechanisms may offer potential strategies to preserve NSC functions and promote healthy hippocampal aging.

### 2.2. Mitochondria Dysfunction and Metabolic Alterations

Similar to other somatic stem cells, adult NSCs exhibit distinct energy demands depending on their activation state. Several studies suggest that quiescent NSCs derive their energy primarily from glycolysis and fatty acid oxidation (FAO), with little reliance on mitochondrial oxidative phosphorylation (OxPhos), which is the main energy source of active NSCs [32,44,45]. Yet, recent studies showing that pyruvate import into mitochondria and OxPhos are required for the maintenance of NSC quiescence suggest that OxPhos is more important in quiescent NSCs than commonly thought [46]. In addition, mitochondria of NSCs undergo metabolic and morphological changes during the transition between quiescent and active states [32,44,45]. Recent studies have highlighted the critical role of these changes as active regulators of NSC fate, rather than being passive adaptations to changing energy demands. Perturbation of mitochondrial metabolic rewiring, for example through deletion of YME1L, compromises hippocampal NSC self-renewal and pool maintenance [47]. Similar outcomes including premature depletion of NSCs, defective neurogenesis, and cognitive impairments were observed upon forced fragmentation of mitochondria by deleting the structural protein MFN1/2 [48]. This study revealed that mitochondrial dynamics play an upstream regulatory role in NSC fate decisions, with enhanced fusion promoting self-renewal and fragmentation favoring neuronal commitment and differentiation. This is achieved through mitochondrial-to-nuclear retrograde signaling, which triggers a dual gene expression program suppressing self-renewal while promoting differentiation [48]. It involves a fission-induced increase in oxidative metabolism and elevated ROS, which serves as signaling mechanism for the stabilization of Nrf2—an antioxidant transcription factor—controlling genes involved in neuronal differentiation, redox response, and Notch signaling [48]. In addition to ROS, mitochondria produce intermediate metabolites which can influence stem cell fate via epigenetic remodeling of nuclear chromatin (see below), including 2-oxoglutarate, acetyl-CoA, and NADH [49]. 2-oxoglutarate serves as a substrate for DNA and histone methyltransferases, while acetyl-CoA acts as co-factor for lysine acetyltransferases, which reverse the activity of NAD^+^-sensing sirtuin-family deacetylases by catalyzing the acetylation of histones and other proteins [49]. Sirtuins, as metabolic sensors, possess antioxidant and anti-proliferative effects and have been implicated in the maintenance of hippocampal NSCs [50,51]. These insights, along with the association of mitochondrial dysfunction with various neurodevelopmental disorders [52], emphasize the critical role of mitochondria in preserving the functional capacity of adult NSCs.

With advancing age, mitochondria become increasingly dysfunctional, characterized by an accumulation of mitochondrial DNA (mtDNA) mutations, impaired dynamics and respiratory function, and elevated production of ROS, all of which are interrelated and potentially contribute to impaired NSC homeostasis [50,53,54,55,56]. It is conceivable that the excessive generation of ROS, coupled with diminished antioxidant capacities in aged NSCs, contributes to the depletion of NSCs observed during aging, as demonstrated in superoxide dismutase-deficient mice or hematopoietic stem cells [57,58]. Moreover, suprathreshold accumulation of mtDNA damage and concomitant activation of sirtuins via an elevated NAD^+^/NADH ratio may represent a mechanism underlying the astrogliogenic disposal of NSCs in the aged DG [5,50].

Recent studies point to a crucial role of lipid metabolism in the regulation of NSCs, although its impact during aging remains to be investigated. In the young SGZ, build-up of lipids via fatty acid synthase-dependent de novo lipogenesis is necessary for the activation of quiescent NSCs and NSC proliferation [59], while their degradation by FAO is required for maintaining the pool of quiescent NSCs [60,61]. Lipids are molecules with manifold functions, ranging from energy storage, membrane assembly, and intra- and inter-cellular communication to gene expression and epigenetic regulation [62]. Therefore, imbalances in lipid metabolism may contribute to NSC aging in many ways. Since FAO operates in the mitochondrial matrix, it is likely to be affected by age-related mitochondrial dysfunction. This may contribute to perturbed proliferation of hippocampal NSCs and eventually depletion of the NSC pool, as shown after inhibition of FAO in young NSCs [60]. Likewise, age-related impairments in cellular waste disposal mechanisms such as autophagy may lead to shifts in the lipid composition of aged NSCs. Future studies need to clarify how these metabolic pathways and intracellular lipid composition change with age, what specific metabolites affect NSC functions, and which changes in lipid homeostasis may contribute to age-related decline in the NSC population.

Nutrient-sensing pathways, such as the insulin/IGF-1, adenosine monophosphate-activated protein kinase, and NAD^+^-dependent sirtuin pathways, play critical roles in the regulation of adult NSCs and their aging. This involves the previously described FoxO transcription factors acting downstream of the insulin/IGF-1 pathway, which regulate various detoxification processes as a function of nutrient availability and cellular redox state [63]. Excess nutrients and loss of FoxO promote the activation and accelerate the depletion of hippocampal NSCs [37], supporting the idea that downregulation of the insulin/IGF-1 pathway is beneficial for long-term maintenance of quiescent NSCs. This is corroborated by studies showing that suppression of insulin/IGF-1 signaling or dietary restriction delay the age-related decline in NSCs [64,65,66,67]. Moreover, deletion of PTEN, an inhibitor upstream of insulin/IGF-1 signaling that triggers the activation of FoxO3, results in the depletion of the hippocampal NSC pool by increasing proliferation and terminal astrocytic differentiation [4,68].

Sirtuins are metabolic sensors activated by NAD^+^ during cellular energy depletion. Sirt1 has been implicated in NSC homeostasis, acting as epigenetic repressor of the Hes1 promoter, a key effector of Notch signaling [67]. Conditional deletion of sirtuin 1 leads to proliferation and rapid exhaustion of NSCs, suggesting a role in maintaining NSC quiescence [51]. Others have implicated sirtuin 1 in the neuron/astrocyte fate choice during differentiation of embryonic and adult NSCs [51,69]. Thus, sirtuins appear to take center stage in metabolic control of adult NSC homeostasis, acting as a nutrient-responsive regulator of NSC self-renewal and differentiation. Although this needs to be confirmed experimentally, it seems highly plausible that the age-related depletion of the NSC pool is driven by the concomitant decline in NAD^+^ and sirtuin concentrations [70].

### 2.3. DNA Damage and Epigenetic Alterations

To sustain cell-specific gene expression and NSC homeostasis throughout life, it is essential to maintain the stability, integrity, and tightly regulated expression of DNA. A growing number of studies show that DNA is continuously damaged during aging, which, together with the reduced capacity of repair mechanisms, contributes to the progressive decline in stem cell potential [71]. In addition, aged NSCs experience dysregulation of epigenetic patterns that are critical for maintaining their stem cell features and fate, including changes in DNA methylation, histone modifications, and chromatin remodeling [72,73]. In this section, we briefly summarize the available evidence linking DNA damage and epigenetic drift to the functional decline of aged hippocampal NSCs, and refer to other reviews for a more detailed discussion of their possible contributions to stem cell aging [53,74,75,76,77].

DNA damage is one of the hallmarks of organismal aging and a leading cause of functional decline in stem cells. DNA damage in NSCs can result from genotoxic stressors of external and internal origin, including toxic metabolites and ROS, and due to their capacity for self-renewal, replication errors, and telomere attrition [53,77]. To counteract the accumulation of mutations, cells have a complex network of cellular processes that are activated in response to such damage, collectively known as the DNA damage response. These include DNA repair, cell cycle arrest, or, if the damage is too severe for repair, apoptosis, all aimed at maintaining genomic integrity and preventing the transmission of damaged DNA to progeny. When attempts to repair DNA lesions fail and cells become senescent, they acquire an inflammatory phenotype including the secretion of pro-inflammatory cytokines [53,78]. This may not only be detrimental to the damaged NSCs itself, but may also spread to surrounding cells and cause chronic inflammation in the niche, which could contribute to premature aging of neighboring yet unscathed NSCs and their progeny [40,53].

In vitro studies have shown that NSCs derived from aged brains carry multiple genomic alterations in the form of deletions or loss of heterozygosity [79]. Others have observed substantial changes in DNA repair pathways indicative for genotoxic stress which already appear in early stages of hippocampal NSC aging [8]. Limited research has explored the implications of DNA damage in NSCs, with most of the available data derived from SVZ-NSCs. There, irradiation-induced DNA damage results in a typical DNA damage response, including inflammatory cytokine secretion, proliferative arrest, and premature differentiation, which, however, recovers in the long-term through activation of quiescent NSCs [80,81,82]. Hippocampal NSCs are likely to react similarly to DNA damage, since irradiation of the adult DG results in a severe but reversible impairment of neurogenesis [83]. The reversibility of the effect suggests that NSCs are differently vulnerable depending on their state, with quiescent NSCs being better protected from genotoxic insults than their cycling counterparts, similar to what has been observed in hematopoietic stem cells [84]. This idea is further supported by studies in the early postnatal DG, which harbors highly proliferative developmental NSCs that become defective and subsequently deplete upon radiation-induced DNA damage [85]. In line with a higher exposure of dividing NSCs to genotoxic stressors, recent single-cell transcriptome analyses revealed an increased expression of DNA damage response genes in active versus quiescent NSCs in the SVZ, which is independent of age [86].

Damage also occurs in mtDNA, even at a much higher rate than in the nucleus [87]. As mtDNA encodes 13 core OxPhos polypeptides, energy metabolism and respiratory capacity are severely impaired when mtDNA mutations accumulate, which is particularly important for high energy-demanding cells such as active NSCs [54]. Additionally, suprathreshold accumulation of mtDNA mutations leads to excessive production of ROS, which furthers damage to nuclear and mtDNA, reinforces inflammation, and impacts NSC fate decisions and self-renewal [50,53,76]. Wang et al. [50] showed that mice lacking the mtDNA repair protein 8-oxoguanine DNA glycosylase exhibit a shift in their differentiation pattern towards the astrocytic lineage due to the spontaneous accumulation of mtDNA damage. This not only highlights the importance of repair mechanisms to preserve critical mitochondrial functions but also provides a mechanism for the aging-related disposal and astrocytic differentiation of hippocampal NSCs [5].

In addition to preserving the genetic code itself, tight regulation of the epigenetic landscape is critical for maintaining NSC identity and switching between different states and fates in response to environmental signals. This occurs on multiple interconnected layers, including DNA methylation-induced gene silencing, control of gene accessibility by histone modifications and pioneer transcription factors, and the spatial organization of chromatin and nuclear architecture [88]. According to recent findings, erosion of the cell type-specific epigenome may actually be one of the causes of NSC aging [73,75]. As recently shown, aged hippocampal NSCs display a decreased expression of histone lysine demethylases [8], enzymes that modify the accessibility of regulatory gene regions by removing methylation marks from histone tails. Although this requires further investigation, the resulting accumulation of abnormal histone methylation may lead to dysregulation of gene expression programs essential for homeostasis and self-renewal of NSCs, and thus be a cause of increased NSC quiescence in old age. Others observed tremendous age-related DNA methylation changes in the DG including hypomethylation of CpGs and hypermethylation at CpHs [89]. Many of these, including those at neurogenesis-associated gene loci, could be reversed by environmental enrichment, an intervention that stimulates adult neurogenesis, illustrating the close relationship between extrinsic factors, epigenetic regulation, and NSC plasticity [89]. Recently, Tet2, which catalyzes the conversion of 5-methylcytosine to 5-hydroxymethylcytosine (5hmC), was discovered as a possible epigenetic regulator of NSC aging [90]. Both Tet2 expression and 5hmC levels were observed to decrease in the aged DG. The neurogenic deficit of old mice could be mimicked by downregulating Tet2 in young animals, whereas reinstatement of Tet2 levels by overexpression or heterochronic parabiosis replenished the NSC pool and promoted neurogenesis in the aged DG. Methylated CpGs are bound by other epigenetic modifiers, such as the methyl-CpG-binding domain protein 1 (MBD1), which is expressed by NSCs [91]. This binding triggers methylation of histone H3, resulting in transcriptional repression of several target genes including that encoding fibroblast growth factor 2 (FGF-2) [92]. MBD1 deficiency was found to increase proliferation and reduce neuronal differentiation of hippocampal NSCs, accompanied by a repression of lineage differentiation genes and up-regulation of astrocytic genes [91]. Taken together, these data demonstrate the importance of homeostasis in the epigenetic landscape for maintaining NSC identity and shape during aging and raise the possibility of rejuvenating neurogenic capacity by resetting the epigenome through environmental or pharmacological interventions. Of note, chromatin regions associated with metabolic and transcriptional functions bound by key transcription factors promoting quiescence lose accessibility in aged NSCs, suggesting a novel mechanism of age-related NSC dysfunction [73].

Pioneer transcription factors are key regulators of cell fate transitions due to their unique ability to access closed chromatin, and thereby initiate reprogramming of silent genes [93,94]. This allows other regulatory proteins, including transcription factors, chromatin, and histone modifiers, to access chromatin and assemble into activating or repressive regulatory complexes [94]. One such pioneering factor is Ascl1, which is required for activation of quiescent NSCs and establishes an open and permissive chromatin state at genes involved in neuronal differentiation [10,95,96,97]. When Ascl1 was depleted in adult hippocampal NSCs, their behavior resembled that of aged NSCs, which exhibit increased quiescence and decreased propensity to activate [11]. This implies that decreasing levels of Ascl1, as observed in aged NSCs, drive age-associated changes in NSC dynamics and force them into deeper quiescence. However, the extent to which this involves Ascl1’s pioneer activity warrants further investigation. Sox2, a transcription factor involved in self-renewal and maintenance of developing and adult NSCs, also has pioneer activities [98,99,100]. A recent report suggests a critical role for Sox2 in establishing long-range chromatin interactions in NSCs that control the activity of key cell proliferation genes [99]. Others proposed a role of Sox2 in establishing a permissive chromatin state at methylated regulatory regions of neurogenic genes in hippocampal stem and progenitor cells (NSPCs), enabling the activation of neuronal differentiation programs once differentiation cues are received [101]. Moreover, Sox2 cooperates with nuclear structural proteins such as nucleoporins to control the transcriptional landscape of adult NSPCs [102]. Finally, Sox2 has been shown to interact with histone acetylases to promote proliferation [100]. Taken together, this evidence establishes Sox2 as a critical player in self-renewal and fate control of NSCs. Given these functions and the fact that Sox2 is downregulated in the aged DG [25,103], it is reasonable to assume that aberrant Sox2 function contributes to NSC aging.

In addition to their role as a barrier to the segregation of toxic molecules during NSC division, components of the nuclear envelope such as lamins help to anchor chromatin and control gene expression through interaction with epigenetic modifiers [104,105,106]. During aging, lamin B1 expression decreases in hippocampal NSCs, resulting in a reshaping of the nuclear architecture [39,40,107]. Conditional deletion of lamin B1 in NSCs impairs their proliferation and induces their differentiation, which is accompanied by an upregulation of quiescence genes such as Bmp4 and Id4, indicating repression of these genes by lamin B1 [39,42]. Restoration of lamin B1 levels in the aged DG promotes NSC proliferation and neurogenesis [42]. Yet, it remains to be determined whether these observations are due to the role of lamins as epigenetic regulators of stem cell programs or to the accumulation of deleterious molecules due to a dysfunctional barrier. The fact that lamin B1 deletion leads to detachment of lamin-associated chromatin from the nuclear envelope, redistribution of chromatin, and increased activation of histone marks [39,106] suggests that NSC dysfunction in aging is due, at least in part, to lamin B1-associated epigenetic mechanisms.

Overall, the accumulation of DNA damage and epigenetic changes in hippocampal NSCs lead to multiple challenges, such as DNA repair damage, activation of senescence pathways, inflammation, dysregulated gene expression, and thus are primary drivers of NSC aging. Understanding these damage mechanisms in NSCs may offer promising targets for interventions to promote healthy aging in the hippocampus and preserve cognitive functions, especially because loss of epigenetic information is a reversible cause of aging [108].

### 2.4. Cellular Senescence

Cellular senescence represents a state of irreversible cell cycle arrest and an endpoint of the aging-related molecular mechanisms discussed before [109]. It functions as a homeostatic mechanism that inhibits the replication of damaged cells, thereby preventing further expansion of damaged cells [109]. However, the accumulation of senescent cells has detrimental effects when it comes to aging. As a result of successive cell-intrinsic alterations, senescent cells undergo shifts in their secretome towards a more inflammatory state known as senescence-associated secretory phenotype (SASP) [53,77,110]. The secretion of SASP factors during aging contributes to damage in the surrounding cells, creating a more hostile environment and aggravating inflammation, ultimately resulting in a decline in cellular and tissue functionality [109].

Senescence is characterized by specific molecular alterations that can be utilized as markers for identifying senescent cells. These comprise heightened β-galactosidase activity, increased expression of cyclin-dependent kinase inhibitors, reduced levels of lamin B1, and the accumulation of DNA damage [111,112,113]. Importantly, these very characteristics are also observed in aged hippocampal NSCs, demonstrating the increasing senescence of these cells with age [40,114,115,116]. Moreover, at least a few of these alterations appear to be causally linked to NSC dysfunction in aging, such as the upregulation of the cyclin-dependent kinase inhibitor p16INK4a [116,117]. However, experimental manipulation of p16INK4a levels in young and aged NSCs revealed that p16INK4a, instead of inducing senescence, prevents aged quiescent hippocampal NSC from being activated by neurogenic stimuli, implying a role in NSC pool maintenance [116]. On the other hand, a causal relationship between the upregulation of p19ARF and NSC senescence has been demonstrated, albeit only in the SVZ and in a highly artificial senescence accelerated mouse-prone 8 model [118]. The same study proposed alterations in epigenetic regulation as mechanism underlying the accumulation of p19ARF in aged NSCs, specifically the dysregulation of histone deacetylation [118].

Epigenetic changes are increasingly recognized as hallmark of senescent cells among the various cell-intrinsic mechanisms that contribute to cellular senescence [109,119]. One of them is the previously discussed reduction in lamin B1 [40,43,120], which has been linked to a state of deeper quiescence and transformation of aged NSCs into astrocytes [5,39,41,42]. Another senescence-associated epigenetic modifier is the PIWI-like RNA-mediated gene silencing 2 (Piwil2), an endoribonuclease crucial for the biogenesis and function of PIWI-interacting RNAs [121]. Suppression of Piwil2 in NSPCs derived from the postnatal DG induces senescence and astrocytic differentiation, mimicking the situation in aged NSCs whose Piwil2 levels are strongly depleted [121]. Others revealed an epigenetic Plagl2-Ascl1 signaling pathway at the core of hippocampal NSC activation, whose disruption participates in NSC senescence [122]. They showed that the loss of Plagl2 in aged NSCs contributes to reduced accessibility of Ascl1 chromatin and, thus, impairs their self-renewal capacity [10,11,95,96,97,122]. Intriguingly, this study revealed that a combination of Plagl2 induction and inhibition of the pro-aging protein Dyrk1a is sufficient to permanently rejuvenate senescent hippocampal NSCs that are otherwise resistant to manipulation of either of these proteins alone or other senescence markers such as p19ARF [122]. Further research is needed to fully comprehend the implication of these mechanisms in cellular senescence and the aging process.

Altogether, evidence suggests a direct contribution of senescence to the aging-associated impairments of NSCs. The complex interplay between various hallmarks of NSC aging, including the connection between epigenetic alterations and cellular senescence, presents intriguing research areas for further investigation. Furthermore, there is a significant gap regarding the specific molecular mechanisms underlying NSC senescence in the aged DG, underscoring the need for dedicated research to this area. Addressing these gaps is crucial for identifying interventions that can effectively rejuvenate aged NSCs and mitigate the decline in hippocampal neurogenesis and cognition [123].

## 3. Neurogenic Niche

**Figure 3:**
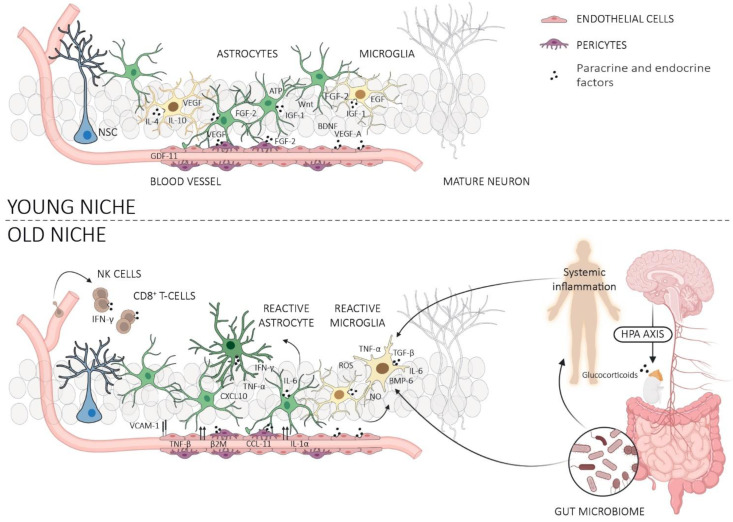
The neurogenic niche and systemic environment as extrinsic regulators of NSC aging. In addition to neurons and their precursor cells, the hippocampal niche is composed of astrocytes, microglia, and a dense network of microvessels which collectively supply NSCs with a range of paracrine, juxtracrine, and systemic cues. With age, glia, endothelial cells, and pericytes release less neurotrophic factors that support NSC proliferation and neuronal differentiation in young mice (FGF-2, IGF-1, EGF, VEGF, ATP, Wnt, BDNF). On the other hand, levels of factors impeding the functions of NSCs increase. Aged astrocytes and microglia adopt a reactive phenotype in which they secrete pro-inflammatory factors (IL-6, TNF-α, IFN-γ, TGF-β), CXCL-10, ROS, NO, and BMP-6. High levels of systemic inflammation, which is partially driven by changes in the gut microbiome, induce the overexpression of VCAM-1 by endothelial cells, which, together with impairments in pericytes, makes the BBB more permeable for blood-derived pro-inflammatory molecules (CCL-11, TNF-β, β2M, IL-1α) and immune cells (NK cells, CD8^+^ T-cells), and thus further exacerbates microglial activation and inflammation in the niche. Moreover, changes in gut microbiota enhance the HPA axis-mediated release of glucocorticoids intothe blood stream, with adverse consequences for NSCs. The figure depicts only factors predominating in the young or increasing in the old neurogenic niche. Abbreviations: β2-microglobulin (β2M), blood–brain barrier (BBB), bone morphogenetic protein 6 (BMP-6), brain-derived neurotrophic factor (BDNF), CXC motif chemokine ligand 10 (CXCL-10), epidermal growth factor (EGF), hypothalamic–pituitary–adrenal (HPA), interferon-γ (IFN-γ), interleukin 6 (IL-6), natural killer (NK), nitric oxide (NO), transforming growth factor-beta (TGF-β), tumor necrosis factor-α (TNF-α), vascular cell adhesion protein 1 (VCAM-1), vascular endothelial growth factor (VEGF).

Adult NSCs reside in a specialized microenvironment which is composed of NSCs and their progeny, as well as parenchymal cells, such as astrocytes, microglia, and endothelial cells from the microvasculature that irrigates the SGZ. These cellular components and the signals released by them play important roles in regulating NSC homeostasis and hippocampal neurogenesis. Recent studies have shown that both the composition of the niche as well as the molecular properties of niche cells undergo significant changes with aging, and that these changes promote the progressive attrition of NSCs via non-cell-autonomous mechanisms (Figure 3).

### 3.1. Astrocytes

Astrocytes are the most abundant glial cell type in the adult DG and perform a variety of important functions from regulating ion and neurotransmitter homeostasis to nutrient supply to releasing neurotrophins and controlling the BBB. In the DG, astrocytes are in close contact with adult NSCs and regulate their behavior through secreted signals and direct cell–cell contacts [124,125,126]. Here, we will focus on factors released or presented by local astrocytes that are known to control NSC behavior and discuss how their dysregulation contributes to the decline in NSC functions and adult hippocampal neurogenesis with aging.

Among the molecules released by astrocytes are FGF-2, VEGF, IGF-1, ATP, and D-serine, which are well known to promote NSC proliferation, both in vitro and in vivo [127,128,129,130,131,132,133,134]. As part of the neurovascular unit, astrocytes also participate in the activity-dependent uptake of such factors from the blood, as has been shown for IGF-1 [135,136]. By releasing vasoactive molecules and matrix metalloproteinase 9-activating factors, astrocytes increase the blood flow rate and promote the cleavage of IGF-1 from its carrier insulin-like growth factor binding protein 3, thereby increasing the bioavailability of circulating IGF-1 in the brain [135,136]. Once there, astrocytes take up the perivascular IGF-1 by their end-feet and transfer it to neighboring cells, such as NSCs and neurons [137,138,139]. Wnt-3 is another factor released by astrocytes that influences the behavior of adult NSCs [140,141,142]. Until now, three Wnt signaling pathways have been discovered downstream of the principal Wnt receptor Frizzled (Fzd): the canonical Wnt/β-catenin pathway and two β-catenin-independent pathways including the planar cell polarity pathway (Wnt/planar cell polarity (PCP) pathway) and the Wnt/Ca^2+^ pathway [143,144]. Of these, the canonical pathway, through which Wnt-3 predominantly acts, has received the most attention in the field of adult neurogenesis. With few exceptions [145], most studies suggest that Wnt/β-catenin signaling induces pro-neural genes and neuronal differentiation in uncommitted progenitor cells and promotes the division of neuronally committed progenitor cells [140,142,145,146,147,148]. Its role in NSCs, which express components of the Wnt/β-catenin pathway [142,149,150], is less clear. In vivo data suggest that baseline activity of the pathway is not required for maintaining the balance between quiescence and activation of NSCs, nor for preserving their stem cell properties [142]. In contrast, stimulation of Wnt/β-catenin signaling by ablation of Wnt antagonists DKK-1 or sFRP-3 in mice resulted in an activation of radial glia-like NSCs [148,151], suggesting that the pathway acts in a dose-dependent manner on NSCs. This is supported by in vitro studies showing that quiescent NSCs become activated by low Wnt/β-catenin stimulation but differentiate in response to high stimulation [142]. The same study found that active NSCs, in contrast to their quiescent counterparts, basically cease to proliferate after Wnt/β-catenin stimulation and instead differentiate, indicating that the response of NSCs to Wnt/β-catenin stimulation further depends on their activation state.

Increasing evidence suggests an important role of direct interactions between astrocytes and NSCs for maintaining NSC homeostasis. Astrocytes in the adult DG express membrane-bound factors such as the Notch ligand Jagged-1 and Ephrin-B2 [152,153]. NSCs in turn express Notch-1 and high levels of the Notch effector gene Hes5 [22,154]. The importance of Notch signaling in maintaining NSC quiescence was demonstrated by ablation of Notch ligands from niche cells or effector genes from NSCs, all of which led to a transient increase in NSC proliferation followed by depletion of the NSC population in the long-term [154,155,156,157]. In contrast, the interaction of Ephrin-B2 presented by astrocytes with Ephrin-B4 receptors on NSCs instructs their neuronal differentiation, probably due to a β-catenin-dependent upregulation of pro-neural genes Ascl1 and Neuro-D1 [152,158]. Hence, astrocytes regulate NSC homeostasis not only through direct release of factors, but also indirectly through their interaction with other cells in the niche and shuttling of systemic factors into adult neurogenic niches.

Recent advancements in single-cell omics technologies revealed that aging can lead to profound changes in the expression of genes that control astrocytic functions [32,159,160], including those involved in the regulation of NSC homeostasis. For example, aged astrocytes secrete fewer trophic factors such as ATP, FGF-2, VEGF, and IGF-1 and thus contribute to the decline in NSC proliferation in the aged DG [161,162,163]. The astrocytic expression of Wnt-3 and the number of Wnt-3 secreting astrocytes are also diminished with aging, further exacerbating the age-related decline in hippocampal neurogenesis [141,164]. By co-culturing aged NSPCs with young or aged primary astrocytes, others demonstrated that aged astrocytes impair the differentiation of NSCs into neurons [141]. This impairment could be reversed by lentiviral overexpression of Wnt-3 in the aged astrocytes, suggesting that the age-related deficit in astrocytic release of Wnt-3 contributes to the decline in NSC differentiation and neurogenesis in the aged hippocampus [141].

Another characteristic of aged astrocytes is the acquisition of a reactive phenotype with hypertrophy of the soma and processes, and substantial shifts in gene expression towards a pro-inflammatory secretory phenotype [159,162,165,166]. Many of the secreted factors, including IL-6, TNF-α, and IFN-γ, are known to impair NSC proliferation and fate determination [86,126,167], and may thus contribute to the age-related impairments in hippocampal neurogenesis and cognition.

Because of their role in regulating and maintaining the BBB, astrocytes help to prevent the uncoordinated invasion of blood-derived factors and immune cells into the surrounding brain parenchyma [160,168]. However, the release of pro-inflammatory factors from aged, reactive astrocytes (IL-6, TNF-α) impairs BBB function and permeability [25,26,169,170,171,172,173]. This could even be exacerbated by the diminished release of Wnts from aged astrocytes, which are crucial for end-feet and BBB integrity at the neurovascular unit [174]. Moreover, aged astrocytes produce increased amounts of chemokines such as CXCL10, which acts as chemoattractant for peripheral immune cells and facilitates the adhesion of T-cells to the luminal surface of the brain vasculature [175]. Aged astrocytes might therefore be involved in the infiltration of IFN-γ expressing T-cells into neurogenic niches and thus indirectly contribute to the increased quiescence of aged NSCs [167]. Although further research is needed, these data provide strong evidence that the age-related dysregulation of astrocytes contributes to leakage of the BBB, allowing harmful compounds and inflammatory cells to enter the hippocampal neurogenic niche, which may ultimately impair NSC functions or cause their death [25,173,176,177].

In conclusion, while enhancing astrocytic function holds promise in sustaining NSC functions and adult hippocampal neurogenesis in aging brains, further research is necessary to fully comprehend the role of astrocytes in NSC homeostasis and aging. For example, additional in vivo studies are needed to unravel the specific contributions of astrocyte-induced Wnt/β-catenin signaling and non-canonical pathways such as the Wnt/PCP pathway, which maintains NSC quiescence in the SVZ [178]. Similarly, the aging-related shifts in Notch signaling and their significance in the context of stem cell aging warrant further investigation. A deeper understanding of the intricate complexity of astrocytes will undoubtedly provide novel insights into their role in NSCs and NSC aging [179].

### 3.2. Microglia

Microglia are the brain´s resident immune cells. With functions ranging from tissue surveillance and phagocytosis to the secretion of inflammatory and trophic molecules, they are critical players in niche homeostasis. In the DG, microglia phagocytize newborn cells undergoing apoptosis [180], and thus protect the niche from harmful contents of dead cells. Interestingly, the beneficial effects of microglial phagocytosis go far beyond the engulfment and elimination of apoptotic cells. As shown recently, phagocytosis of newly born cells triggers a microglial secretome that limits NSC proliferation and hippocampal neurogenesis, suggesting its involvement in a negative feedback loop that maintains homeostasis along the neurogenic lineage [181].

Apart from their phagocytic role, microglia release a plethora of growth factors and cytokines with functions in adult hippocampal neurogenesis [182,183,184]. Depending on the biological context and their functional state, microglia exhibit complex and dynamic roles in the control of adult NSCs, which may be either beneficial or detrimental for their functions [183,184]. Under physiological conditions, microglia are in a homeostatic state in which they secrete factors that stimulate (FGF-2, EGF, IGF-1) [185], but also repress the proliferation of NSCs and instead consolidate NSC quiescence (TGF-β, BMP-6) [176,186]. During tissue surveillance, microglia constantly receive and respond to signals from their environment, which induce a wide spectrum of functional states and secretory phenotypes [184,187]. When exposed to anti-inflammatory cytokines such as those from T-helper type 2 cells, microglia adopt an anti-inflammatory phenotype in which they release cytokines (IL-4, IL-10) and growth factors such as IGF-1 that promote the proliferation and differentiation of NSCs [188,189,190,191]. At the other end of the spectrum, microglia acquire an inflammatory, potentially cytotoxic phenotype in response to pro-inflammatory signals or pathogens. This state is characterized by the release of pro-inflammatory cytokines such as TNF-α and IL-6, which may drive NSCs into deeper quiescence and impair their survival [188,189,192,193,194].

Aging is accompanied by substantial changes in the morphology and molecular landscape of microglia, which compromise their capacity for homeostatic functions. As consequence of the chronic inflammation that occurs during aging, aged microglia display a persistent state of activation and adopt an inflammatory phenotype. This is characterized by an increased secretion of pro-inflammatory cytokines, as well as those with typically anti-inflammatory properties (TGF-β, IL-10) [176,194], the levels of which essentially determine whether they act anti- or pro-inflammatory. While TGF-β and IL-10 are anti-inflammatory under physiological conditions, they can exacerbate inflammatory responses when released excessively in situations of sustained inflammation, such as aging [176,194,195]. Additionally, active microglia produce less antioxidants and release reactive factors such as ROS and NO [196], which, along with pro-inflammatory cytokines, can cause cytotoxicity and intensify inflammation in the aged hippocampus. This might impair the BBB’s function, leading to the leakage of systemic factors and immune cells into the niche, which can aggravate the inflammatory response and finally impair NSCs functions [194,196,197]. Moreover, aged microglia produce lower levels of growth factors such as IGF-1 [161,162] and an increased amount of anti-proliferative cues such as BMP-6 [176], which can directly contribute to the dormancy of NSCs in the aged DG [21]. This is indirectly exacerbated by the transformatory action of pro-inflammatory microglia on astrocytes, causing them to adopt an inflammatory-reactive phenotype [86,126,165,167,198]. Notably, Clarke et al. [165] found that mice lacking pro-inflammatory signals, such as IL-1α, TNF, and C1qa, show a decrease in the expression of genes associated with reactive astrocytes. This suggests that targeting these signals could potentially serve as therapeutic intervention to alleviate the excessive inflammation in the aged niche and improve the capabilities of aged NSCs.

Aged microglia lose their dynamic surveillance features and exhibit a decreased phagocytic capacity [199,200,201], caused at least in part by an upregulation of the phagocytosis-inhibitor CD-22 and the accumulation of insoluble, lipofuscin-like inclusions resulting from clearance of over-fragmented myelin [202]. Defective phagocytosis results in the accumulation of toxic compounds in the neurogenic niche and thus more pronounced neuroinflammation, accompanied by an increased activation of microglia and astrocytes [165], infiltration of immune cells from the systemic environment [167,173], and weakening of the BBB [203].

These data expose microglia as profoundly pleiotropic players in the hippocampal stem cell niche that influence stem cell aging both directly and indirectly through modulation of other niche components. Targeting microglial phagocytosis, inflammation, and ROS/NO production may represent promising therapeutic approaches to improve NSC homesotasis and hippocampal neurogenesis during aging. However, further research is needed to fully elucidate the role of microglia in NSCs aging and to identify molecular targets for therapeutic interventions.

### 3.3. Microvasculature, Endothelial Cells, and Pericytes

The DG is irrigated by a dense vascular network which is in close contact with the cells of the niche and supplies them with the necessary energy. Together with pericytes, the endothelial cells lining the walls of this microvasculature are key constitutents of the BBB [204]—a highly specialized interface that controls the molecular and cellular traffic between the circulation and the central nervous system, preventing uncontrolled access of circulating factors and toxins [168,205].

Endothelial cells express a variety of soluble factors, such as cytokines and growth factors, as well as membrane-bound factors involved in juxtracrine signaling that directly regulate the proliferation and fate of adult NSCs [206,207]. One of these factors is the chemokine stromal cell-derived factor (SDF), which was shown to control the activity of NSCs in the SVZ [208]. There, active NSCs are found in close proximity to microvessels expressing SDF, while quiescent NSCs were associated with SDF-negative microvessels [208]. Moreover, endothelial cells express a variety of signals supporting NSC quiescence, such as Notch ligands delta-like protein 4 and Jagged-1, neurotrophin 3, and Ephrin-B2 [209,210,211].

However, little is known about the role of endothelial-derived factors in the maintenance of NSC homeostasis in the SGZ. As astrocytes and microglia, endothelial cells in the DG express pro-neurogenic soluble factors such as VEGF-A and BDNF, which are known to promote hippocampal neurogenesis and cognitive functions [212,213,214,215,216]. Although the expression of their receptors VEGFR-2 and TrkB on hippocampal NSCs suggests a potential direct influence on NSC behavior [213,215,217], it is not yet clear whether endothelial-derived VEGF and BDNF act directly on hippocampal NSCs, which is questioned by recent studies using VEGF On/Off mice [214]. However, VEGF-A appears to promote NSC activation indirectly via the stimulation of angiogenesis. In this regard, VEGF-induced proliferation of microvessels promotes asymmetric NSC division in the young DG, whereas it expands the NSC pool in the aged DG. Remarkably, these effects are long-lasting and increase the proportion of active NSCs without accelerating their exhaustion, making VEGF a promising candidate for NSC activation in aging [214]. Furthermore, with their specialized barrier and shuttling function, endothelial cells are responsible for the transport of molecules into or out of the neurogenic niche. This includes, for example, the pro-neurogenic circulating factor IGF-1, whose bioavailability in the niche is enhanced by the concerted action of endothelial cells and astrocytes [135,136,139]. Apart from that, endothelial cells actively maintain homeostasis within the niche, exemplified by their ability to translocate lactate, a byproduct of glucose metabolism, into the bloodstream [218].

Single-cell RNA sequencing revealed significant transcriptional changes in aged hippocampal endothelial cells [172,218]. In fact, endothelial cells undergo the highest transcriptional changes during aging compared to other parenchymal cells, primarily due to their exposed location and sensitivity to the aging systemic milieu [86,172]. Many of the genes that are upregulated in aged endothelial cells are associated with inflammation, senescence, and oxidative stress [172]. This is evident, for example, for TGF-β [172,219], a cytokine that triggers cell cycle arrest and apoptosis of cycling NSCs [219,220]. The age-related molecular and structural alterations of endothelial cells lead to the deterioration of the BBB, which becomes more permeable with aging [26,170,171,177]. Consequences range from dysregulated nutrient and ionic fluxes to plasma leakage and extravasation of inflammatory cytokines and leukocytes [25,169,170,171]. For example, upregulation of VCAM-1 on aged DG-endothelial cells in response to increased systemic inflammation facilitates leukocyte tethering to their luminal side, which exacerbates vascular inflammation and microglial activation. This ultimately leads to a reduction in NSC proliferation and hippocampus-related cognitive impairments [221]. Trafficking through the aged BBB is further disturbed by shifts in transcytosis of plasma proteins as well as a decrease in pericyte coverage in the DG. Finally, the shift from ligand-specific receptor-mediated to nonspecific caveolar transcytosis in aged endothelial cells allows the entry of neurotoxic proteins (albumin, fibrinogen) into the neurogenic niche [177], with deleterious effects on the NSC pool.

Pericytes are specialized cells wrapping around endothelial cells that play a crucial role in controlling blood vessel density and various aspects of their function, including vascular tone and blood flow [222,223]. As key components of the BBB, they regulate the permeability for macromolecules and ensure BBB integrity through their modulatory influence on endothelial and astroglial gene expression [222]. While nothing similar has yet been reported for the DG, evidence suggests a regulatory influence of pericytes on the proliferation and differentiation of NSPCs in the SVZ, mediated indirectly via control of cytokine expression in astrocytes and other cells of the niche [224,225]. In addition, pericytes are a source of several NSC-effective factors (FGF-2, TNF-α, VEGF) [226,227], suggesting a direct regulatory influence on NSC behavior and aging, although detailed studies are pending.

Hippocampal pericyte aging has been shown to compromise the integrity of the BBB, resulting in the extravasation and accumulation of immune cells and macromolecules that induce cell death and worsen cognitive function [223]. As mentioned earlier, this may entail activation of microglia and an increased inflammatory response, which ultimately compromise the function of NSCs [197,198,225,227]. Further research is necessary to discern the full spectrum of effects of pericytes on hippocampal NSCs and their implication in the age-related decline in neurogenesis.

NSCs in turn actively interact with blood vessels to regulate local blood flow and survey changes in the systemic environment [125,228,229]. The latter has been demonstrated in the DG, where terminal arborizations of NSCs wrap around microvessels in the inner molecular layer to form perivascular end-feet that resemble and cooperate with those of astrocytes [126,229]. These specialized neurovascular units allow hippocampal NSCs to directly sense circulating factors despite a fully functional BBB [126]. NSCs of the SVZ establish similar contacts with the vasculature by sending projections that terminate on niche capillaries. There, the activation of NSCs triggers an increase in capillary tone and local blood flow, which is mediated via the release vasoactive factors from their terminal end-feet that bind to purinergic receptors on adjacent pericytes [230]. Whether NSCs in the DG affect blood flow in a similar way remains to be seen. However, the data available so far point to a reciprocal communication between NSCs and the microvasculature, revealing NSCs as direct players in neurovascular coupling and direct sensors of systemic signals.

Although much remains unknown, available data along with the central position of the microvasculature at the interface between the systemic milieu and the brain suggest that its structural and functional transformation in the aging niche is critically involved in the loss of niche homeostasis, the decline in NSC functions, and the activation of microglia observed with aging.

## 4. Systemic Milieu

In addition to being regulated by their local microenvironment, NSCs are also exposed to systemic signals entering the neurogenic niche via the circulation. These include blood components (plasma proteins, immune cells), circulating soluble factors (hormones, cytokines, growth factors), and extracellular vesicles, among others, many of which originate from distant regions of the body. In this role, blood is an important mediator of lifestyle factors such as exercise or diet, which could be implemented to modify neurogenesis. Aging leads to a number of changes in the systemic milieu that can have significant impacts on NSC functions. One of the most prominent changes is an increase in chronic low-grade inflammation throughout the body, termed as “inflammaging”. Moreover, aging is accompanied by an increase in oxidative stress, hormonal changes, and alterations of the microbiome, all of which are interconnected. While potentially any systemic signal reaching the niche has the capacity to affect NSCs, few have been studied for their effects on the NSC population and during aging. This section discusses how systemic influences may affect NSCs and their niche and highlights the gaps that will definitely need further research.

### 4.1. Blood-Derived Factors and Extracellular Vesicles (ECVs)

The most convincing evidence for an influence of systemic factors entering the neurogenic niche via the circulation comes from heterochronic parabiosis and plasma transfer experiments [200]. Exposure of young mice to old blood decreased the proliferation and number of NSPCs, and ultimately impaired hippocampal neurogenesis, learning, and memory, whereas the opposite was observed in DGs of old heterochronic parabionts [25,230]. Similar observations were made when human multipotent hippocampal progenitor cells were grown in the presence of serum from donors of different ages [231]. Among the factors mediating the pro-aging effects of aged plasma in the DG niche are CCL-11—an eosinophil-recruiting chemokine—and β2M—a component of type 1 major histocompatibility complex). Both increase with age and disturb NSPC proliferation and neurogenesis in vivo and in vitro, although their actual effects specifically on NSCs remain to be clarified [25,169]. Cyclophilin-A—an enzymatically active cytokine frequently cited as a pro-aging factor for hippocampal neurogenesis and cognition—does not appear to act at the NSC level [232].

In addition, aged blood is enriched in pro-inflammatory factors due to the accumulation of senescent cells secreting pro-inflammatory cytokines (IL-1α, TNF-β), the accumulation of pro-inflammatory tissue damage, and the impaired autophagy response throughout the body [233,234]. Many of them are likely to have indirect effects on NSCs through modulation of the cells in the niche. For example, plasma from old mice induces the expression of VCAM-1 on endothelial cells, in a similar way to IL-1α and TNF-β, pushing microglia into a reactive state that forces NSCs into deeper quiescence [221,235]. This is partially countered by the concomitant increase of circulating anti-inflammatory cytokines such as Il-4, which induces the secretion of IGF-1 and other pro-neurogenic factors from microglia, whose plasma concentrations decrease with age [189,236]. Moreover, the aging-related chronic inflammation leads to activation of the hypothalamic–pituitary–adrenal axis, which augments the secretion of glucocorticoids [233,237]. As recently shown, NSCs expressing glucocorticoid receptors are more resistant to age-related decline than those lacking them, suggesting a protective role of glucocorticoids in these cells [238]. This study shows that glucocorticoids prevent NSC activation through epigenetic programming of cell cycle and Wnt signaling genes, thereby preserving a reserve of quiescent NSCs beyond adolescence. However, excessive amounts of glucocorticoids in the elderly are suppressive to NSCs by impairing not only their proliferation but also their survival [239,240]. Sex hormones, on the other hand, are downregulated with aging [241], with potentially detrimental effects on NSCs. Although they do not autonomously affect basal proliferation of NSCs, they act synergistically with growth factors such as EGF and FGF-2 to enhance their proliferative activity [242]. In addition, estrogens and androgens prevent oxidative stress-induced growth reduction [242]. Considering that growth factor levels decrease with age and oxidative stress levels increase, the shortage of sex hormones may exacerbate the deleterious aspects of NSC aging. An age-related downregulation is also observed for growth differentiation factor 11, a bone morphogenic protein involved in blood vessel maintenance [243]. This may contribute to the deterioration of vascular function and blood flow observed in aged neurogenic niches [244], ultimately impairing NSC nutrient supply and function. Accordingly, systemic administration of growth differentiation factor 11 to old mice improved vascular remodeling, neurogenesis, and cognitive functions in the DG, and thus is a promising rejuvenation approach to mitigate various aspects of stem cell aging [245,246,247].

In addition to soluble factors, blood contains ECVs produced by almost every cell type in the body [248]. ECVs transport various cargo including proteins, nucleic acids, lipids, and metabolites that potentially influence NSCs. Evidence suggests that ECVs from different sources participate in cell cycle regulation and influence NSC proliferation [249,250,251]. Both the content and number of ECVs change with aging [252]. Aged ECVs play important roles in transmitting aging signals from the periphery to the neurogenic niches, which can induce inflammation, ROS accumulation, genomic instability, and mitochondrial, lysosomal, and proteasome dysfunction, with deleterious consequences for NSCs [253]. For example, aged ECVs shuttle less Klotho mRNA, whose protein product supports NSC proliferation and neurogenesis in the DG, and thus may contribute to the increased quiescence of aged NSCs [254,255]. Moreover, senescent cell-derived ECVs, recently uncovered as key players in the SASP, may promote senescence of NSCs and induce genomic instability [253,256,257]. All this suggests that ECVs are important contributors to NSC homeostasis. Yet, much remains unknown, although the ECV field is continuously expanding, and more work is required to detail their impact on NSC functions and aging.

### 4.2. Gut Microbiome

Another emerging topic is the regulation of NSCs by the gut microbiome. The gut microbiota produces various metabolites and signaling molecules that can alter the functions of NSCs either directly or indirectly. For instance, they can stimulate the production of neurotrophins in the hippocampus, act as upstream regulators of the host immune response and have profound effects on the hypothalamic–pituitary–adrenal axis [258]. Accordingly, studies suggest that microbiome dysbiosis in aging may cause a decrease in the proliferation and differentiation of NSCs as well as an increase in neuroinflammation through modulation of microglia and hypothalamic-pituitary-adrenal axis-mediated cortisol release [259]. This is worsened by the fact that the reduction of beneficial microbiota increases systemic inflammation and oxidative stress [259,260], which further drives NSC aging (Figure 3). However, direct evidence regarding the involvement of the gut microbiome in NSC functions is currently limited. Thus, it will be important to unravel the interactions between gut microbiota, NSCs, and the aging process to exploit their therapeutic potential. This is all the more because the gut microbiome is highly amenable to interventions, such as dietary modification, pro and prebiotics, and fecal transfer, providing numerous opportunities to improve NSC functions and plasticity in aged brains.

### 4.3. Peripheral Immune Cells

Aging of hematopoietic stem cells themselves appears to have no impact on NSC aging, although it impairs hippocampal neurogenesis and cognition [232]. However, there are several lines of evidence for beneficial and detrimental roles of the peripheral immune system in neurogenesis, involving both innate and adaptive immune cells. Several studies imply that homeostatic neurogenesis in young neurogenic niches depends on peripheral and central nervous system-specific CD4^+^ T-cells, and that these cells support the proliferation and differentiation of NSPCs and cognitive function [190,261,262,263]. However, aged neurogenic niches are infiltrated by cytotoxic CD8^+^ T-cells as revealed in single-cell transcriptome studies in humans and mice [167,173]. In the SVZ, these cells are clonally expanded and differ from those in blood in that they express distinct T-cell receptors and high levels of IFN-γ [167]. This leads to strong IFN-γ responses in NSCs and other IFN-γ receptor expressing cells such as microglia and endothelial cells, suggesting widespread contributions to niche aging. As demonstrated by Dulken et al. [167], the T-cell-derived IFN-γ has deleterious effects on NSC proliferation, which was later supported by IFN-γ loss-of-function experiments that led to recovery of NSC functions in the aged SVZ [86]. Although relevant studies are still pending, these mechanisms probably also apply to the aged DG, which exhibits a comparable accumulation of CD8^+^ T-cells [173]. Moreover, the aged DG displays an accumulation of NK cells, which even exceeds that of other immune cells [173]. Apparently, these NK cells are responsible for the elimination of senescent neuroblasts, by which they are attracted and activated via their senescence-associated secretome. It remains to be determined whether these cells also affect NSCs.

### 4.4. Glymphatic System and Meningeal Lymphatic System

The glymphatic system and meningeal lymphatic system have been recently recognized as players in niche homeostasis and potential determinants of NSC functions. The glymphatic system is an astroglia-dependent system of perivascular tunnels with important roles in waste clearance from the interstitial space, including metabolites and pathogenic proteins [264,265]. The meningeal lymphatic system has similar functions in draining macromolecules, but also in immune cell trafficking between the central nervous system and cervical lymph nodes [266,267]. Aging affects the function of both systems, which results in the accumulation of cytotoxic molecules in the brain [266,268]. This contributes, for example, to the accumulation of amyloid-β in the aged hippocampus, which has been associated with NSC senescence and reduced NSC proliferation and neuronal differentiation, both in vitro and in vivo [269,270,271]. Although the direct relationships remain to be investigated, these results provide preliminary evidence for an involvement of the glymphatic and meningeal–lymphatic systems in the age-related impairments in NSC homeostasis. Modulation of their drainage and immune functions in the elderly opens new avenues to improve dysfunctional neurogenesis and cognition in old age.

Altogether, these findings provide evidence for the involvement of the systemic milieu in NSC aging, either directly or through modulation of other cells in the niche, such as microglia or endothelial cells. Further research is warranted to determine the precise interplay between specific systemic factors and NSC aging. It is reasonable to assume that future studies on the interaction between the gut microbiota and NSCs will lead to readily implementable, promising therapeutic approaches, particularly given the ease of access and manipulability of the microbiome.

## 5. Conclusions

Extensive research in recent years has significantly advanced our knowledge of the mechanisms underlying NSC aging and age-related decline in neurogenesis, although much remains obscure. Central to this decline is an escalating impairment of the NSC pool, characterized by increased quiescence, altered lineage specification, and progressive depletion of NSCs. The mechanisms underlying NSC aging in the DG are complex and multifactorial. Over the course of their life, NSCs accumulate several defects, including a failure to maintain a healthy proteome, metabolic alterations, DNA damage, and epigenetic drift. It is now recognized that, in addition to intrinsic mechanisms, extrinsic changes in the NSC niche and systemic environment are the primary contributors to NSC aging, and that these mechanisms are not mutually exclusive, but rather interrelated and interacting with each other.

To safeguard the NSC pool from depletion, it is vital to gain a comprehensive understanding of the cellular and molecular mechanisms regulating NSCs and their aging. The advent of innovative new techniques such as single-cell RNA sequencing and spatial transcriptomics holds immense potential for unravelling the full complexity of the mechanisms involved in the declining capacities of NSCs during aging. Other technologies, such as CRISPR-Cas or adenovirus-mediated gene transfer, enable diverse types of gene function screens, facilitating the exploration of molecular interdependencies and their impact on NSC aging. Understanding the mechanisms that control NSC functions and their aging holds potential for the identification of novel therapeutic targets to either slow the aging process or rejuvenate aged NSCs, thereby enhancing the regenerative and cognitive capacities of the aging hippocampus. Preventively, simple interventions with few side effects, such as diet or exercise, are particularly promising. Curatively, it becomes more difficult, as the interventional activation of NSCs usually leads to premature exhaustion and accelerated depletion of the pool [51,154,272,273,274]. However, recent findings that have been able to identify targets whose manipulation increases the self-renewal of NSCs in the aged DG without accelerating their depletion (VEGF, combination of Plagl2 with anti-Dyrk1a) give cause for optimism [123,216].
